# Explainable multilingual and multimodal fake-news detection: toward robust and trustworthy AI for combating misinformation

**DOI:** 10.3389/frai.2025.1690616

**Published:** 2025-12-10

**Authors:** Rohini Jadhav, Vishal Meshram, Amol Bhosle, Kailas Patil, Sital Dash, Shrikant Jadhav

**Affiliations:** 1 Bharati Vidyapeeth (Deemed to be University) College of Engineering, Pune, India; 2 Vishwakarma Institute of Technology, Pune, India; 3 MIT Art, Design and Technology University, Pune, India; 4 Vishwakarma University, Pune, India; 5 San Jose State University, San Jose, CA, United States

**Keywords:** fake news detection, misinformation and disinformation, multilingual dataset, explainable artificial intelligence, hybrid deep learning architecture, adversarial robustness, social-media analysis

## Abstract

Fake-news detection requires systems that are multilingual, multimodal, and explainable—yet the majority of the existing models are English-centric, text-only, and opaque. This study introduces two key innovations: (i) a new multilingual–multimodal dataset of 74,000 news articles in Hindi, Gujarati, Marathi, Telugu, and English with paired images, and (ii) Hybrid Explainable Multimodal Transformer Fake (HEMT-Fake) that integrates text, image, and relational signals with hierarchical explainability. The architecture combines transformer embeddings, a convolutional neural network–bidirectional long short-term memory (CNN–BiLSTM) text encoder, residual network (ResNet) image features, and graph sample and aggregate (GraphSAGE) metadata, all of which are fused via multi-head attention. Its explainability module unites attention, Shapley Additive exPlanations (SHAP), and local interpretable model-agnostic explanations (LIME) to provide token-, sentence-, and modality-level transparency. Across four languages, HEMT-Fake delivers a ~ 5% Macro-F1 improvement over Cross-Lingual Language Model with RoBERTa (XLM-R) architecture and Multilingual Bidirectional Encoder Representations From Transformers (mBERT), with gains of 7–8% in low-resource languages. The model achieves 85% accuracy under adversarial paraphrasing and 80% on artificial intelligence (AI)-generated fake news, halving robustness losses compared to baselines. Human evaluation reveals that 82% of explanations are judged to be meaningful, confirming transparency and trust for fact-checkers.

## 1 Introduction

### 1.1 The global challenge of fake news

The global information ecosystem is undergoing rapid transformation, driven by the increasing dominance of digital and social-media platforms. While these platforms democratize content creation and dissemination, they also amplify the reach of misinformation and disinformation, often without adequate verification. Early data-mining perspectives on fake-news detection established hybrid models that capture social propagation and content features (Shu et al., 2017; Castillo et al., 2011), paving the way for transformer-based architectures that now dominate the field. The consequences are profound: misinformation can distort electoral outcomes, incite social unrest, and erode trust in scientific and healthcare institutions (Lazer et al., 2018; Tandoc et al., 2018). During the coronavirus disease 2019 (COVID-19) pandemic, false narratives regarding vaccines and treatments spread virally, at times with more traction than evidence-based information (Shahi et al., 2021) The ability to automatically detect fake news at scale has thus become not only a technological challenge but also a societal imperative.

### 1.2 The rise of AI-generated misinformation

The landscape of misinformation is further complicated by advances in generative artificial intelligence (AI). Large Language Models (LLMs) such as Generative Pre-Trained Transformer 4 (GPT-4), Gemini, and Large Language Model Meta AI (LLaMA) are capable of producing linguistically coherent, contextually relevant, and stylistically adaptive narratives at scale (Ji et al., 2023). Similarly, image and video generation models such as Stable Diffusion and DeepFakes enable the creation of visually convincing synthetic content. This convergence of text–image manipulation poses unprecedented challenges for fact-checkers and automated systems alike (Zellers et al., 2019). Importantly, generative models allow adversaries to create misinformation tailored for specific linguistic, cultural, or political contexts, making multilingual and multimodal detection more urgent than ever.

### 1.3 Limitations of current fake-news detection systems

Although significant research has been conducted on fake-news detection, existing approaches exhibit critical shortcomings:

1. *Monolingual bias*: the majority of the datasets and detection models are English-centric (Shu et al., 2017; Alam et al., 2021). Low-resource and code-mixed languages remain underexplored, limiting the global applicability of detection systems.
2. *Insufficient multimodal fusion*: many studies treat text and images independently or use simplistic late fusion strategies (Zhou et al., 2020). However, fake news often relies on cross-modal inconsistency (misleading captions paired with unrelated or manipulated images).
3. *Opaque decision-making*: transformer-based architectures such as Bidirectional Encoder Representations From Transformers (BERT) and Cross-Lingual Language Model with RoBERTa (XLM-R) deliver state-of-the-art accuracy but are widely criticized as black boxes (Lu et al., 2023). Without clear justifications, stakeholders such as journalists, policymakers, and the public may distrust AI predictions.
4. *Adversarial vulnerability*: even minor perturbations (synonym substitutions, paraphrasing) significantly degrade performance (Yang et al., 2022). Recent studies show that GPT-generated fake articles can bypass detectors entirely (Jawahar et al., 2023).

### 1.4 Why do multilingual, multimodal, and explainable AI (XAI) matter?

A robust fake-news detection system must address three interrelated priorities:

- *Multilingual robustness:* in multilingual societies, misinformation circulates in regional languages, often mixed with English or transliterated into Latin scripts. Models trained exclusively on English fail to capture cultural idioms and code-switching behaviors (Dementieva et al., 2023; Yigezu et al., 2024).
- *Multimodal integration:* misinformation is increasingly leveraging multimodal artifacts such as memes, manipulated videos, or misattributed images. Ignoring visual modalities leads to incomplete detection pipelines (Xu et al., 2024; Choi and Kim, 2024).
- *Explainability:* trustworthy AI requires interpretable outputs. Black-box predictions without transparent reasoning hinder adoption by journalists, fact-checkers, and policymakers. Advanced methods, such as Shapley Additive exPlanations (SHAP) and local interpretable model-agnostic explanations (LIME), can reveal feature contributions, while hierarchical attention can highlight key tokens and sentences (Nwaiwu et al., 2025).

Together, these considerations underscore that future research must move beyond unimodal, monolingual, and opaque models to embrace hybrid, explainable, and resilient architectures.

### 1.5 Motivations for this study

This study is motivated by the pressing need for practically deployable systems for detecting fake news. While prior research has demonstrated strong accuracy in benchmark settings, practical deployment requires balancing accuracy, robustness, and transparency. Consider, for example, a fact-checking newsroom in India where misinformation spreads across Hindi, Marathi, Gujarati, and Telugu. A monolingual English model would be ineffective; a black-box multimodal model would be mistrusted; and a non-robust system would fail against adversarial paraphrases. This scenario exemplifies why an effective solution must simultaneously support multilingual generalization, multimodal fusion, and human-understandable explanations.

### 1.6 Research gap and contributions

*Research gaps identified:*

*RG1*: Absence of large-scale, multilingual multimodal datasets reflecting authentic, code-mixed misinformation.

*RG2*: Poor cross-lingual transferability beyond high-resource languages.

*RG3*: Limited multimodal integration with weak detection of cross-modal inconsistencies.

RG4: Explanations restricted to token-level saliency, with little validation of their usefulness to humans.

*RG5*: Lack of resilience to adversarial and generative AI-driven misinformation.

*Contributions of this study:*

1. Dataset Innovation: A curated dataset of ~74,000 articles across four Indian languages (Gujarati, Hindi, Marathi, and Telugu), incorporating multimodal and adversarially perturbed samples.
2. Architectural Innovation: Proposal of Hybrid Explainable Multimodal Transformer Fake (HEMT-Fake), which integrates multilingual embeddings (XLM-R), convolutional neural network–bidirectional long short-term memory (CNN–BiLSTM) encoders, residual network (ResNet)-based image embeddings, and graph sample and aggregate (GraphSAGE) propagation signals.
3. Explainability Innovation: A hybrid module combining hierarchical attention, SHAP, and LIME to generate token-, sentence-, and modality-level explanations.
4. Robustness Innovation: Training with adversarial paraphrases, back-translations, and GPT-generated fakes to enhance resilience.
5. Evaluation Contribution: Comprehensive experiments including zero-shot cross-lingual testing, multimodal ablations, robustness evaluation, and *human-centered validation* of explanations with journalists and students.

### 1.7 Article organization

The remainder of the article is structured as follows: Section 2 provides a critical review of prior literature on fake-news detection, focusing on multilingual, multimodal, and explainable approaches. Section 3 describes the dataset. Section 4 details the proposed methodology. Section 5 presents experimental settings. Section 6 reports results. Section 7 discusses implications, and Section 8 concludes with future directions.

## 2 Literature review

### 2.1 Overview and linking to research gaps

This review addresses five persistent research gaps (RGs) identified in Section 1:

(RG1) limited multilingual coverage and cross-lingual robustness,

(RG2) inadequate multimodal integration and cross-modal inconsistency detection,

(RG3) shallow or unvalidated explainability,

(RG4) lack of adversarial testing and robustness to LLM-generated fakes, and

(RG5) dataset limitations (absence of multilingual, multimodal, adversarial, and rationale-annotated corpora).

For each gap, representative studies (2017–2025) are critically compared, methodological constraints are highlighted, and their implications for the proposed HEMT-Fake framework are discussed.

### 2.2 RG1—multilingual coverage and cross-lingual robustness

*State of the art.*

Multilingual transformer backbones—XLM-R, mBERT, RemBERT, and mT5—remain foundational for cross-lingual misinformation tasks (Devlin et al., 2019; Conneau et al., 2020; Liu et al., 2019). An early credibility analysis by Castillo et al. (2011) and subsequent follow-ups by Shu et al. (2020) established text-centric baselines that were later extended into multilingual settings. Recent efforts include hybrid summarization and retrieval-augmented multilingual models (Alghamdi et al., 2024a; Alghamdi et al., 2024b; Khandelwal et al., 2024) and low-resource evaluations in African and Indian contexts (Arega, 2025; Al-Zahrani and Al-Yahya, 2024). Recent efforts include hybrid summarization and retrieval-augmented multilingual models (Alghamdi et al., 2024a; Alghamdi et al., 2024b; Khandelwal et al., 2024) and low-resource evaluations in African and Indian contexts (Arega, 2025; Al-Zahrani and Al-Yahya, 2024). Large-scale multilingual benchmarks—PolyTruth (Gouliev et al., 2025; Macko et al., 2025) and the Macko et al. (2025)—quantify degradation on low-resource and code-mixed data and reveal that even strong encoders struggle with dialectal variation and transliteration.

*Critical comparison.*

- Han (2022) and Dementieva et al. (2023) confirmed gains from multilingual encoders but avoided transliteration or noisy social-media code-mixing.
- Gouliev et al. (2025) and Mohtaj et al. (2024) highlighted low-resource gaps yet lacked multimodal or explanation annotations.
- Regional datasets, such as those studied by Arega (2025) and Al-Zahrani and Al-Yahya (2024), underscore domain biases absent from global corpora.

*Shortcomings relative to RG1.*

The majority of the multilingual systems optimize for scale but not realism: they ignore (a) tokenization under code-mixing and transliteration, (b) multimodal or cross-lingual visual cues, and (c) human-validated explanations. Hence, multilingual models remain brittle when claim and evidence languages differ. HEMT-Fake addresses this by integrating multilingual transformers with CNN/BiLSTM branches to reinforce cross-lingual semantics.

### 2.3 RG2—multimodal integration and cross-modal inconsistency detection

*State of the art.*

Multimodal fake-news detection has progressed from late-fusion to cross-modal reasoning. Early multimodal baselines (Pérez-Rosas et al., 2018; Ruchansky et al., 2017) introduced textual–visual pairings. Modern systems, such as Multimodal Adaptive Graph-Based Intelligent Classification (MAGIC) (Xu et al., 2024) and Tri-Transformer Bootstrapping Language–Image Pretraining (TT-BLIP) Choi and Kim (2024), employ graph attention and BLIP-style tri-transformers. Robust multimodal frameworks (FKA-Owl Authors, 2024; Lu and Yao, 2025; Li et al., 2024; Zhang et al., 2024) strengthen alignment but rarely target multilingual or adversarial contexts. Nasser et al. (2025) and Chalehchaleh et al. (2024) survey emerging multimodal defenses, while Practical Newsroom Adoption Studies (2024) demonstrate the need for interpretable, operational tools.

*Critical comparison.*

- MAGIC’s graph fusion exploits propagation but presumes high-fidelity alignment and fails under doctored visuals.
- TT-BLIP enhances image–text coherence but remains English- or Chinese-centric.
- Low-resource multimodal datasets (Lekshmi Ammal and Madasamy, 2025; Macko et al., 2025) expand language scope yet lack adversarial perturbations.

*Shortcomings relative to RG2.*

Few systems quantify modality-specific contributions, detect cross-modal contradictions, or sustain performance when image/text quality diverges. HEMT-Fake’s hierarchical attention fusion explicitly models these inconsistencies and supports multilingual visual reasoning.

### 2.4 RG3—Explainability: from attention maps to human-actionable rationales

*State of the art.*

Explainable-AI methods for misinformation detection range from attention visualization to attribution-based and hybrid approaches. Early feature-based transparency models, such as those discussed by Wang (2017), established interpretable linguistic cues for deception detection. Hu et al. (2022a), combine co-attention with knowledge distillation for multimodal reasoning. Panchendrarajan et al. (2024) and Hardalov (2022) review XAI fidelity issues, with a particular emphasis on multilingual rationales. The XPLAINLP (2025) framework extends this line by producing counterfactual and feature-level explanations for multilingual transformer outputs, offering practical templates for fact-checking. X-FRAME (Nwaiwu et al., 2025) similarly integrates XLM-R embeddings with LIME-based attribution, while Muñoz et al. (2024) conduct user studies that confirm that hybrid explanations improve trust—an insight echoed by policy and ethics analyses of automated fact-checking (2024).

*Critical comparison.*

- Attention alone often lacks causal fidelity (Jain and Wallace, 2019).
- LIME and SHAP yield feature importance but are unstable for long multilingual documents (Wang, 2017).
- Hybrid attention + SHAP designs lack systematic human validation or cross-modal transparency.

*Shortcomings relative to RG3.*

Explainability progress remains fragmented—mainly characterized by *post hoc* and unimodal approaches. Gaps persist in (a) integrating hierarchical attention across languages and modalities, (b) producing human-readable rationales, and (c) user–study validation. HEMT-Fake’s explainability module unites attention, SHAP, and LIME with cross-lingual evidence mapping to address these.

### 2.5 RG4—adversarial robustness and LLM-generated misinformation

*State of the art.*

Recent analyses also link the generation of synthetic misinformation to broader issues of hallucination and factual unreliability in large language models (Ji et al., 2023), underscoring the need for adversarial evaluation of detectors trained on content generated by LLM. Transformers are vulnerable to paraphrasing, synonym substitution, and synthetic news (Nakamura et al., 2020; Cui and Lee, 2020). Shu et al. (2020) formalized early adversarial data splits, while Kukkar et al. (2025) and Chalehchaleh et al. (2024) propose adversarial training and perturbation frameworks. FKA-Owl Authors (2024) extends robustness testing to vision–language models.

*Critical comparison.*

- Nakamura et al. (2020) show large drops under paraphrasing, but only in English.
- Jawahar et al. (2023) and related studies demonstrate detector evasion by LLM-generated fakes.
- Recent study on defensive distillation and multilingual adversarial augmentation (Kukkar et al., 2025; Nasser et al., 2025) remains under-evaluated across modalities.

*Shortcomings relative to RG4.*

Adversarial defenses are piecemeal: few assess multilingual, multimodal, and LLM-driven perturbations jointly. HEMT-Fake introduces paraphrasing, back-translation, and LLM-based negative augmentation for resilience testing across languages and modalities.

### 2.6 RG5—dataset limitations: coverage, adversarial examples, and rationale annotations

Benchmark datasets—LIAR, FakeNewsNet, Fakeddit, and CoAID (Cui and Lee, 2020)—underpin the majority of the progress (Shu et al., 2020). Since 2022, new multilingual and multimodal datasets such as Dementieva et al. (2023), Yigezu et al. (2024), Gouliev et al. (2025), Macko et al. (2025), Mohtaj et al. (2024), and the Macko et al. (2025) have emerged, extending coverage across languages and modalities. However, the majority of them still lack integrated adversarial negatives and rationale annotations.

*Critical comparison.*

- Dementieva et al. (2023) and Gouliev et al. (2025) benchmark multilingual retrieval yet lack image and rationale alignment (Gouliev et al., 2025).
- Yigezu et al. (2024) and Lekshmi Ammal and Madasamy (2025) datasets are of low scale and single domain (Lekshmi Ammal and Madasamy, 2025).
- Shared tasks vary in annotation consistency.

*Shortcomings relative to RG5.*

Existing datasets seldom combine (i) multilingual code-mixing, (ii) multimodal pairing, (iii) adversarial perturbations, and (iv) explanation labels. This constrains research on holistic modeling and faithful XAI. HEMT-Fake’s evaluation corpus fills this gap with all four attributes.

### 2.7 Cross-cutting methodological trends and best practices

Recent studies highlight the following:

1. Hybrid architectures combining transformer, CNN, BiLSTM, and Graph Neural Network (GNN) components (MAGIC; FKA-Owl 2024; Lu and Yao, 2025) for multimodal temporal reasoning.
2. Pretrained vision–language backbones (BLIP/CLIP and TT-BLIP) adapted for low-resource multilingual captions (Zhang et al., 2024; Li et al., 2024).
3. Synthetic augmentation with LLMs to craft adversarial negatives (Kukkar et al., 2025; Nasser et al., 2025) while guarding against label leakage.
4. Hybrid XAI pipelines integrating attention + SHAP/LIME with human-validated evaluations (Hu et al., 2022a; Muñoz et al., 2024; Policy/Ethics Analyses of Automated Fact-Checking, 2024).

### 2.8 Synthesis: how the prior study motivates HEMT-fake

Despite progress in multilingual transformers, multimodal fusion, and explainability, no system satisfies *all* operational demands for fact-checking across multilingual, multimodal, and adversarial environments. Prior studies typically optimize a subset—such as language breadth, modality fusion, or explainability—but not all dimensions together.

HEMT-Fake integrates:

- Multilingual transformer backbones with cross-lingual evidence retrieval → addresses RG1 (Alghamdi et al., 2024a; Arega, 2025).
- Multimodal fusion using transformer + CNN + BiLSTM + optional GNN propagation → addresses RG2 (MAGIC; Lu and Yao, 2025).
- Hierarchical explainability combining attention, SHAP, LIME, and evidence retrieval → addresses RG3 (Hu et al., 2022a; Muñoz et al., 2024).
- Adversarial augmentation with paraphrase and LLM-generated negatives → addresses RG4 (Nakamura et al., 2020; Kukkar et al., 2025).
- Evaluation on a new multilingual + multimodal + adversarial dataset with human explanation ratings → addresses RG5 (Mohtaj et al., 2024; Macko et al., 2025).

### 2.9 Concluding remarks on the review

The 2017–2025 literature converges on a key insight: success in fake-news detection depends not only on representational accuracy but on cross-lingual generalization, multimodal reasoning, faithful explainability, adversarial resilience, and human validation. The proposed HEMT-Fake framework operationalizes these five principles, bridging gaps identified across prior studies and aligning with current practical and ethical expectations for deployable fact-checking systems (Practical Newsroom Adoption Studies, 2024; Policy/Ethics Analyses of Automated Fact-Checking, 2024).

## 3 Data description

### 3.1 Scope and sources

To enable reproducible and representative experimentation, a multilingual, multimodal dataset (Patil et al., 2024) was compiled between January and May 2024. The dataset spans five languages—Hindi, Gujarati, Marathi, Telugu, and English—and includes both textual claims and associated images. Sources include:

1. *Fact-checking platforms* (AltNews, BoomLive, Factly, and International Fact-Checking Network [IFCN] members, etc.)—serving as the gold standard for labeling fake vs. real content.
2. *Mainstream news portals* (The Hindu, The Indian Express, BBC Hindi, etc.)—supplying reliable, real news samples.
3. *Social-media posts* (Twitter/X, Facebook public pages, etc.)—candidate fake content validated against fact-checking repositories.

Each sample includes a unique identifier, textual content, metadata (including language, publication date, source Uniform Resource Locator [URL], and category), and image references.

**Figure 1:**
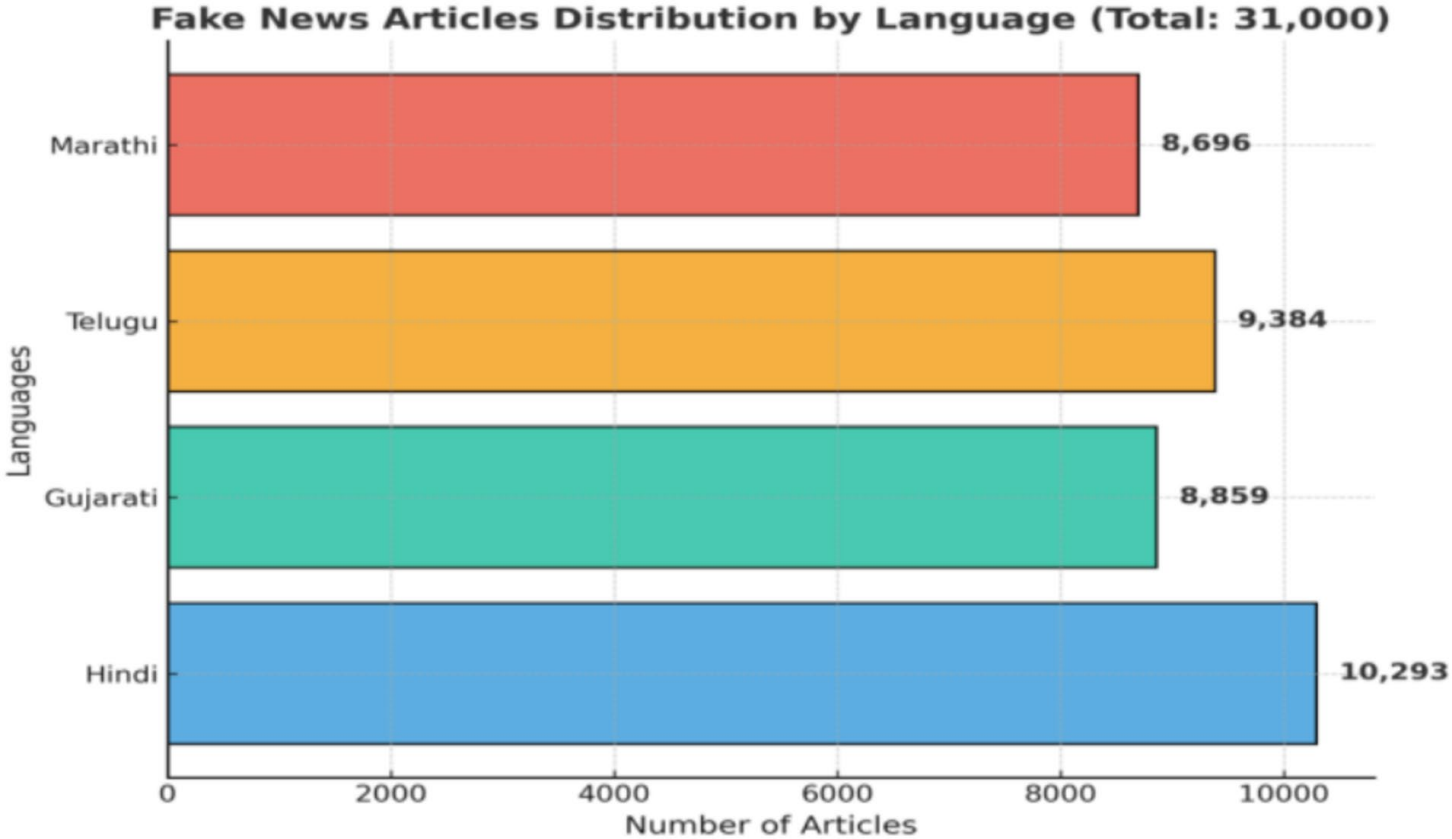
Language-wise dataset distribution. Distribution of fake and real articles across Hindi, Gujarati, Telugu, Marathi, and English, demonstrating multilingual coverage and balanced sampling for cross-lingual robustness.

To illustrate the distribution of multilingual content, we present the language-wise breakdown of fake and real news articles in the dataset. Figure 1 highlights the balanced representation across Hindi, Gujarati, Telugu, Marathi, and English, ensuring that no single language dominates the dataset. This balanced coverage is crucial for developing robust multilingual models that generalize effectively across diverse linguistic contexts.

**Figure 2:**
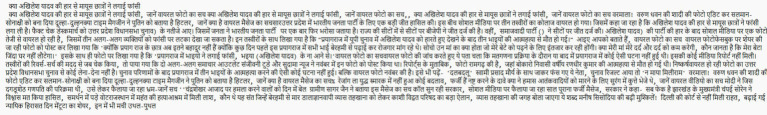
Example of a Hindi article. A representative Hindi news article included in the dataset, annotated as either Fake or Real. The figure illustrates the dataset’s raw structure and the challenges posed by script diversity and linguistic complexity.

In addition to distribution statistics, it is essential to demonstrate the nature of raw multilingual articles included in the dataset. Figure 2 presents a representative Hindi article, showcasing the script, structural format, and annotation label (“Fake or Real”). Including such examples highlights the complexity of real-world data, where articles often contain a mix of linguistic styles, varied sentence lengths, and domain-specific terminology.

**Figure 3:**
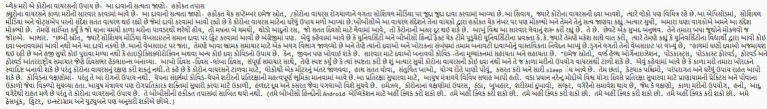
Example of a Gujarati article. A representative Gujarati news article from the dataset, annotated as either Fake or Real. The figure highlights challenges such as script-specific tokenization, code-mixing with English, and domain-specific vocabulary that complicate the detection of multilingual fake news.

To further highlight the dataset’s multilingual nature, Figure 3 illustrates a representative Gujarati news article. Gujarati content in the dataset captures both formal reporting from news portals and informal narratives from social-media platforms. These examples reveal challenges such as script-specific tokenization, mixed use of English and Gujarati words, and domain-specific terms that complicate automated fake-news detection.

**Figure 4:**
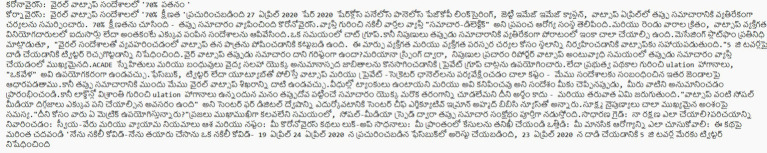
Example of a Telugu article. A representative Telugu news article from the dataset, annotated as either Fake or Real. The figure highlights the linguistic complexity of Telugu, including compound word structures, agglutinative morphology, and frequent code-mixing with English, which makes multilingual fake-news detection particularly challenging.

The dataset also includes a significant portion of content in Telugu, one of the most widely spoken Dravidian languages in India. Figure 4 shows a representative Telugu article, annotated as Fake or Real. Telugu data presents unique challenges for automatic detection, including complex script morphology, agglutinative grammar, and compound word formation. In addition, many Telugu articles demonstrate code-mixing with English, reflecting the real-world writing style found in social-media and online portals.

**Figure 5:**
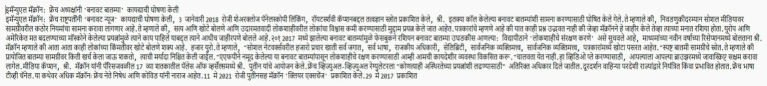
Example of a Marathi article. A representative Marathi news article from the dataset, annotated as either Fake or Real. The figure highlights linguistic and stylistic complexities such as dialectal variation, transliteration of English terms, and shifts between formal and informal registers that complicate automated multilingual fake-news detection.

The dataset also contains substantial content in Marathi, a language with rich inflectional morphology and regional variations. Figure 5 presents a representative Marathi article, annotated as Fake or Real. Marathi articles in the dataset range from formal news reports to colloquial narratives posted on social media. This diversity introduces challenges such as handling dialectal variations, transliterated English words, and stylistic differences between formal and informal registers.

### 3.2 Data collection flow

**Figure 6:**
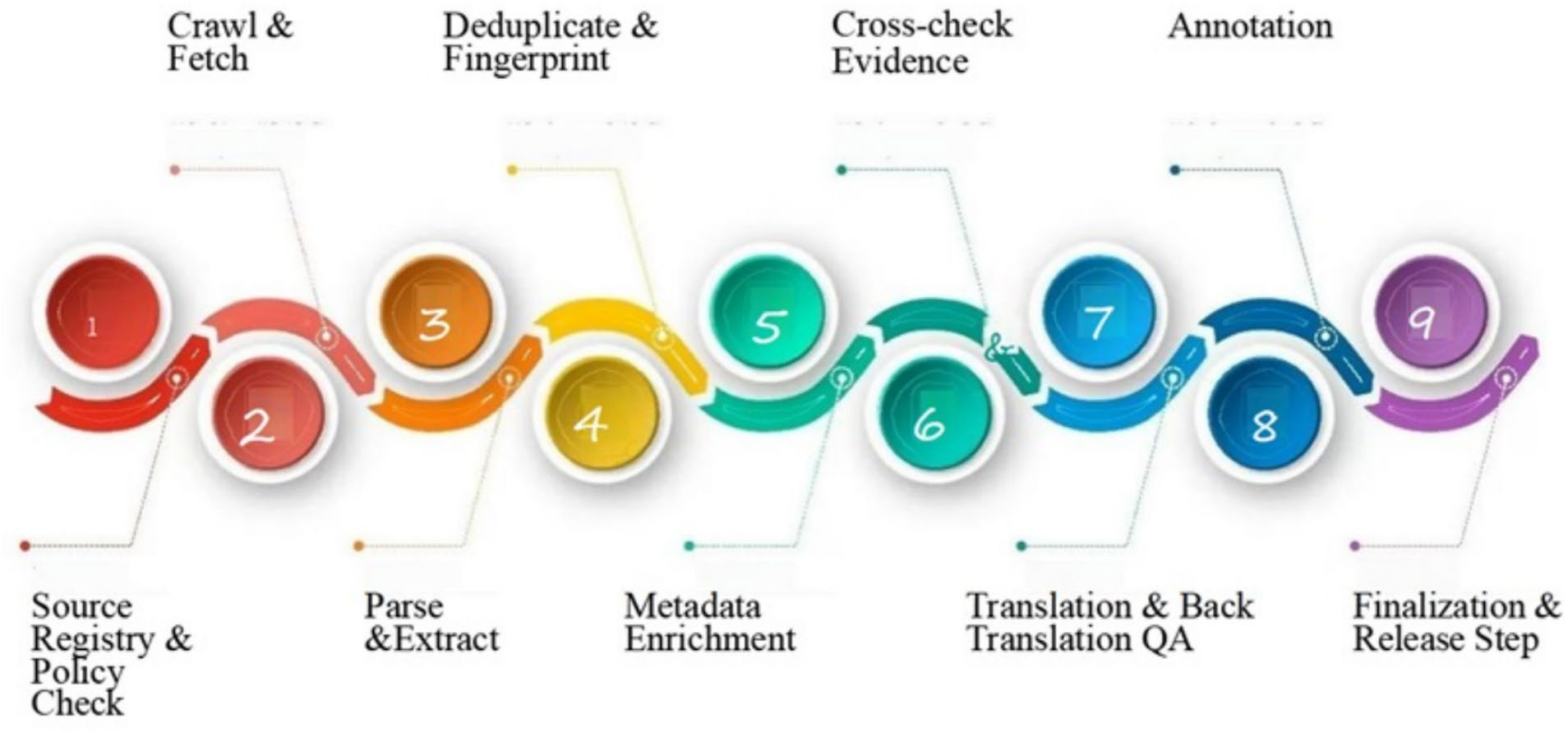
End-to-end data collection and preparation pipeline. The pipeline consists of nine stages: (1) source registry and policy compliance checks, (2) crawl and fetch of candidate articles, (3) parsing and metadata extraction, (4) deduplication and fingerprinting, (5) metadata enrichment, (6) evidence cross-checking with fact-checking repositories, (7) translation and back-translation quality assurance, (8) annotation by trained multilingual annotators, and (9) finalization and release preparation. This process ensures the production of high-quality, ethically compliant, and reproducible multilingual fake-news data.

To provide a visual overview of the dataset development process, Figure 6 depicts the end-to-end pipeline used to construct the multilingual fake-news dataset. The pipeline integrates ethical and legal compliance checks, large-scale crawling, parsing and extraction, deduplication, metadata enrichment, fact-check alignment, translation with semantic quality assurance, multilingual annotation, and final dataset release. This systematic workflow ensures reproducibility, balanced multilingual representation, and transparency in the dataset creation process.

Figure 6 shows the following steps for the dataset development process.

*Step 1: Source Registry and Policy Check.*

All candidate domains were verified for registry information, publication credibility, and licensing policies. Automated crawlers respected robots.txt directives, and sources that prohibited data usage were excluded to ensure legal compliance.

*Step 2: Crawl and Fetch.*

Articles were collected using domain-specific Application Programming Interfaces (APIs), Really Simple Syndication (RSS) feeds, and custom crawlers. Crawling was performed under strict rate-limiting and retry mechanisms to prevent server overload and comply with platform guidelines.

*Step 3: Parse and Extract.*

From each retrieved webpage, the title, body text, images, and relevant metadata (e.g., publication date, author, and URL) were extracted. Non-textual noise, such as advertisements, scripts, and extraneous Hypertext Markup Language (HTML) tags, was discarded.

*Step 4: Deduplication and Fingerprinting.*

To eliminate redundancy, near-duplicate articles were identified using SimHash-based fingerprinting with a similarity threshold of 0.85. Exact duplicates were removed based on canonical URLs and text hashing.

*Step 5: Metadata Enrichment.*

Each article was enriched with additional attributes, including automatic language detection, topical categorization (such as politics, health, and entertainment), and geolocation metadata. This enrichment facilitated downstream analysis and stratified balancing.

*Step 6: Cross-check Evidence.*

Candidate claims were verified against fact-checking repositories, including AltNews, BoomLive, and IFCN-certified platforms. Each item was validated against verified fact-check entries, allowing confident assignment of Fake or Real labels.

*Step 7: Translation and Back-Translation Quality Assurance.*

Non-English articles were translated into English using MarianMT, and semantic fidelity was verified through back-translation. Instances with similarity scores below 0.55 were flagged for human review to maintain translation quality.

*Step 8: Annotation.*

Three trained bilingual annotators independently reviewed each article. Labels (Fake or Real) were assigned following strict guidelines, and disagreements were resolved via adjudication. Inter-annotator agreement reached a substantial level (*κ* = 0.82).

*Step 9: Finalization and release preparation.*

The dataset was anonymized by removing personally identifiable information (PII), assigned unique identifiers, and packaged into a version-controlled release. A public release was prepared, including licensing, documentation, and a metadata manifest.

Figure 6 illustrates this complete end-to-end pipeline. To complement this pipeline, the preprocessing strategies applied after collection are summarized in Table 1, which outlines the cleaning, balancing, and augmentation methods used on the dataset.

**Table 1:** Summary of dataset composition, preprocessing, and balancing across five languages (Hindi, Gujarati, Marathi, Telugu, and English).

Stage	Techniques applied
Text cleaning	Unicode normalization, stopword removal, and transliteration normalization
Duplicate detection	SimHash fingerprinting (threshold 0.85) and canonical URL checks
Noise filtering	Minimum of 50 tokens/article, low-quality translation removal, and corrupted images were discarded
Image preprocessing	Resize to 224 × 224, histogram equalization, and perceptual hashing (pHash)
Class balancing	Stratified sampling, oversampling of the minority, and undersampling of the majority classes
Text augmentation	Synonym replacement, back-translation, paraphrasing (mT5 and Pegasus), adversarial perturbations
Image augmentation	Rotation, flips, Gaussian noise, brightness/contrast adjustment, and cropping/zooming
Cross-modal augmentation	Artificially misaligned text–image pairs to simulate inconsistencies
Quality assurance	Semantic similarity validation, 5% manual spot checks, and version-controlled logs

The table reports the number of instances before and after cleaning, the percentage of Fake or Real labels, and the augmented samples.

### 3.3 Article collection

The first stage of dataset creation is a robust article collection pipeline designed to ingest multilingual content from heterogeneous sources (fact-checking portals, mainstream news outlets, and social-media feeds). This algorithm ensures that the dataset respects legal and ethical constraints while maximizing coverage across languages.

The process begins with a registry of approved sources, where each domain is validated for crawl permissions via robots.txt and licensing terms. Once verified, the crawler fetches articles using RSS feeds, sitemaps, or site-specific APIs. To preserve data quality, the pipeline applies rate-limiting and retry mechanisms to prevent overloading servers or missing content due to transient errors.

Each fetched article is then parsed for metadata (title, author, publication date, text body, and images) and subjected to deduplication using SimHash-based fingerprinting. This step prevents redundancy and ensures that the dataset contains unique entries. The algorithm also records license metadata and stores raw HTML snapshots for reproducibility.

Pseudocode: Algorithm 1—Multilingual Article Collection.

In summary, Algorithm 1 ensures a legally compliant, deduplicated, and metadata-rich corpus that serves as the foundation for subsequent translation, annotation, and classification steps.

**Algorithm 1:**
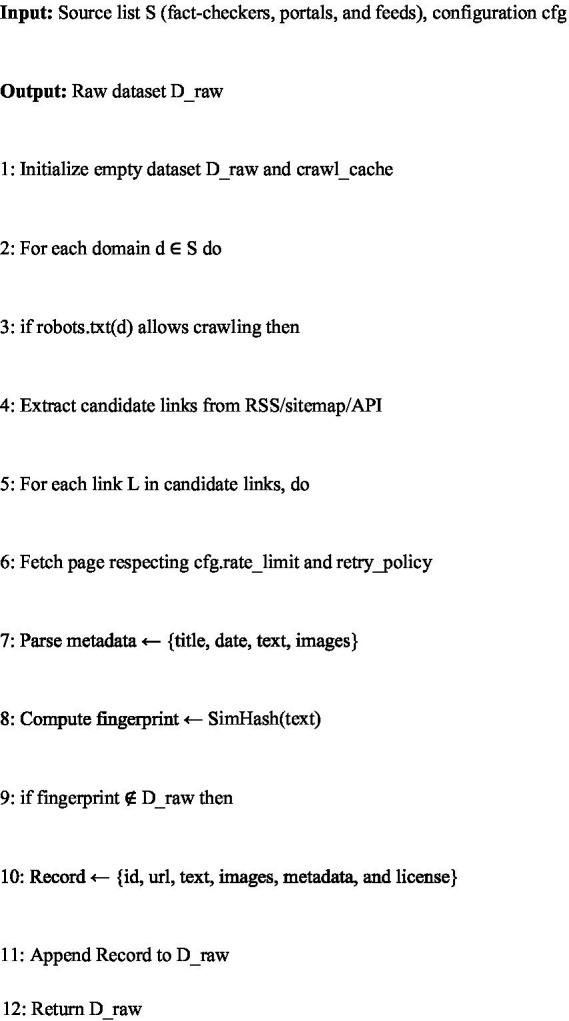
Multilingual article collection and ingestion.

### 3.4 Article translation and normalization

Given the multilingual nature of the dataset, the second stage involves translation and quality assurance to align non-English content into a common pivot language (English). This alignment enables consistent cross-lingual representation learning and facilitates evaluation across multiple languages.

The algorithm begins with language detection using a fastText-based classifier. If the article is already in English, it is stored directly. Otherwise, it is translated into English using MarianMT/Opus-MT (the preferred offline engine) or a fallback API when needed.

To safeguard translation quality, a back-translation step is performed: the translated English text is re-translated into the original language. The original and back-translated texts are then compared using semantic similarity (cosine embeddings) and optional Bilingual Evaluation Understudy/Translation Edit Rate (BLEU/TER) scores. If the similarity exceeds a threshold, the translation is accepted. Otherwise, the article is flagged for human review, where bilingual experts adjudicate translation fidelity.

Each translated article is stored with its original version, pivot translation, back-translation, similarity metrics, and a quality flag. This ensures traceability and transparency in multilingual preprocessing.

Pseudocode: Algorithm 2—Translation, Back-Translation and Quality Assurance (QA).

In summary, Algorithm 2 ensures that the dataset is linguistically aligned, semantically faithful, and quality-controlled, thereby enabling robust multilingual fake-news detection experiments.

**Algorithm 2:**
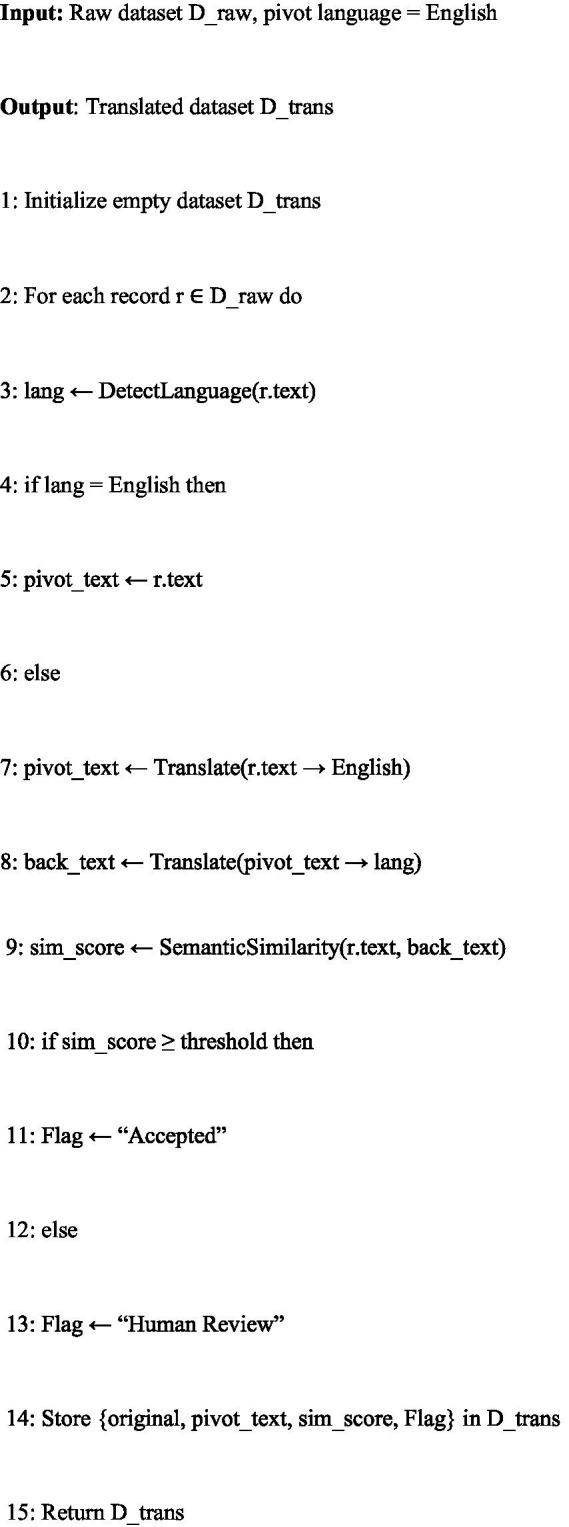
Article translation, back-translation, and QA.

### 3.5 Annotation and quality control

- Annotators: Three bilingual experts per language (linguists and journalists).
- *Label schema:* Fake (verified false), Real (verified true).
- *Inter-annotator agreement: κ* = 0.82, indicating substantial agreement.
- *Consensus:* Disagreements were resolved via adjudication meetings.
- *Image verification:* Reverse-image search and Exchangeable Image File Format (EXIF) metadata analysis to detect manipulations.
- *Adversarial samples:* Synthetic fakes generated via paraphrasing and LLMs were flagged separately for adversarial testing.

### 3.6 Dataset statistics

- *Total articles:* Notably, 74,032 (Fake = 37,232; Real = 36,800).
- *Languages:* Hindi (20,493), Gujarati (17,859), Telugu (18,284), and Marathi (17,396).
- *Images:* ~22,000 paired with text samples.
- *Domains:* Politics (32%), health (24%), environment (12%), entertainment (18%), local issues (14%).
- *Average length:* A total of 245 tokens/article (text), 1.3 images/article (where present).

### 3.7 Cleaning, balancing, and augmentation

To ensure that the dataset is reliable, unbiased, and suitable for multilingual fake-news detection, a systematic multi-stage process was applied to clean, balance, and augment the collected articles. Table 1 presents a structured overview of the preprocessing pipeline designed to ensure high-quality, balanced, and robust multilingual–multimodal data. The cleaning stage involved text normalization, duplicate detection using SimHash, noise filtering, and image preprocessing. The balancing stage applied stratified sampling, oversampling of minority classes, and undersampling of overrepresented categories to maintain a 1:1 ratio between *Fake* and *Real* news within each language. The augmentation stage incorporated a combination of text-based transformations (synonym replacement, paraphrasing, back-translation, and adversarial perturbations), image-level augmentations (rotation, flips, brightness/contrast adjustment, and Gaussian noise), and cross-modal augmentation by intentionally misaligning text–image pairs. Finally, quality assurance checks (semantic similarity validation, manual spot-checks, and version-controlled logs) were applied to guarantee semantic fidelity and reproducibility.

#### 3.7.1 Cleaning and normalization

- *Text cleaning:* All raw text was normalized into the Unicode Transformation Format-8-bit (UTF-8) format to accommodate multilingual characters. HTML tags, scripts, advertisements, emojis, and non-informative tokens were removed. Stopwords were filtered using language-specific stopword lists (Hindi, Gujarati, Marathi, Telugu, and English). Code-mixed and transliterated text was normalized using phonetic matching and transliteration libraries to standardize representation.
- *Duplicate detection:* Near-duplicate entries were removed using SimHash-based content fingerprinting with a similarity threshold of 0.85. Exact duplicates were eliminated by checking canonical URLs and text hashes.
- *Noise filtering*: Articles with fewer than 50 tokens were discarded as they lacked sufficient information for classification. Low-quality translations (semantic similarity score < 0.55 in back-translation checks) were flagged and either corrected through human review or excluded. Corrupted or broken image files were discarded.
- *Image preprocessing:* All images were resized to 224 × 224 pixels. Histogram equalization and color normalization were applied to improve feature extraction. Duplicate or visually identical images were removed using perceptual hashing (pHash).

#### 3.7.2 Class balancing

Class imbalance was addressed to ensure fair learning across Fake and Real categories:

- *Stratified sampling:* Ensured equal representation across the five languages and both classes.
- *Oversampling:* Minority classes (e.g., Gujarati Real articles) were oversampled using data duplication with slight perturbations.
- *Undersampling:* Majority classes (e.g., Hindi Fake articles) were reduced to maintain a balanced 1:1 ratio between classes within each language.
- *Final ratio:* Approximately 50:50 between Fake (37,232) and Real (36,800).

#### 3.7.3 Data augmentation

To increase robustness, especially for low-resource languages, augmentation techniques were applied at both the text and image levels:

- *Text augmentation*

*Synonym replacement:* Randomly replaced content words with synonyms using multilingual WordNet resources.

*Back-translation:* Articles were translated into English and then back to the original language to generate paraphrased variants while preserving the original meaning.

*Paraphrasing:* Transformer-based paraphrasers (mT5 and Pegasus Multilingual) generated semantic variants.

*Adversarial perturbations:* Character-level perturbations (e.g., homoglyph substitution and misspellings) were introduced to simulate adversarial noise.

- *Image augmentation:* Random rotation (±15°), horizontal/vertical flips, and slight Gaussian noise were applied. Brightness and contrast adjustments simulated variable-quality uploads from social media. Cropping and zooming simulated partial screenshots and low-resolution reposts.
- *Cross-modal augmentation:* Misaligned text–image pairs were artificially created (e.g., pairing an image from one article with unrelated text) to train the model to detect cross-modal inconsistencies.

#### 3.7.4 Quality assurance

- Each augmented dataset batch was automatically validated with semantic similarity checks to ensure label consistency.
- Manual spot checks by annotators were performed on 5% of augmented samples to verify quality.
- All augmentation processes were logged, version-controlled, and reproducible via preprocessing scripts.

### 3.8 Dataset availability

The dataset generated and analyzed in this study is an original, multilingual dataset curated by the authors and is publicly available in full. The complete dataset, along with preprocessing scripts and annotation guidelines, can be accessed at Zenodo DOI: 10.5281/zenodo.11408513. The dataset is released under a Creative Commons Attribution–NonCommercial 4.0 International (CC BY-NC 4.0) license, which permits reuse and adaptation for academic research with appropriate citation but prohibits commercial use without explicit permission from the authors.

## 4 Methodology

**Figure 7:**
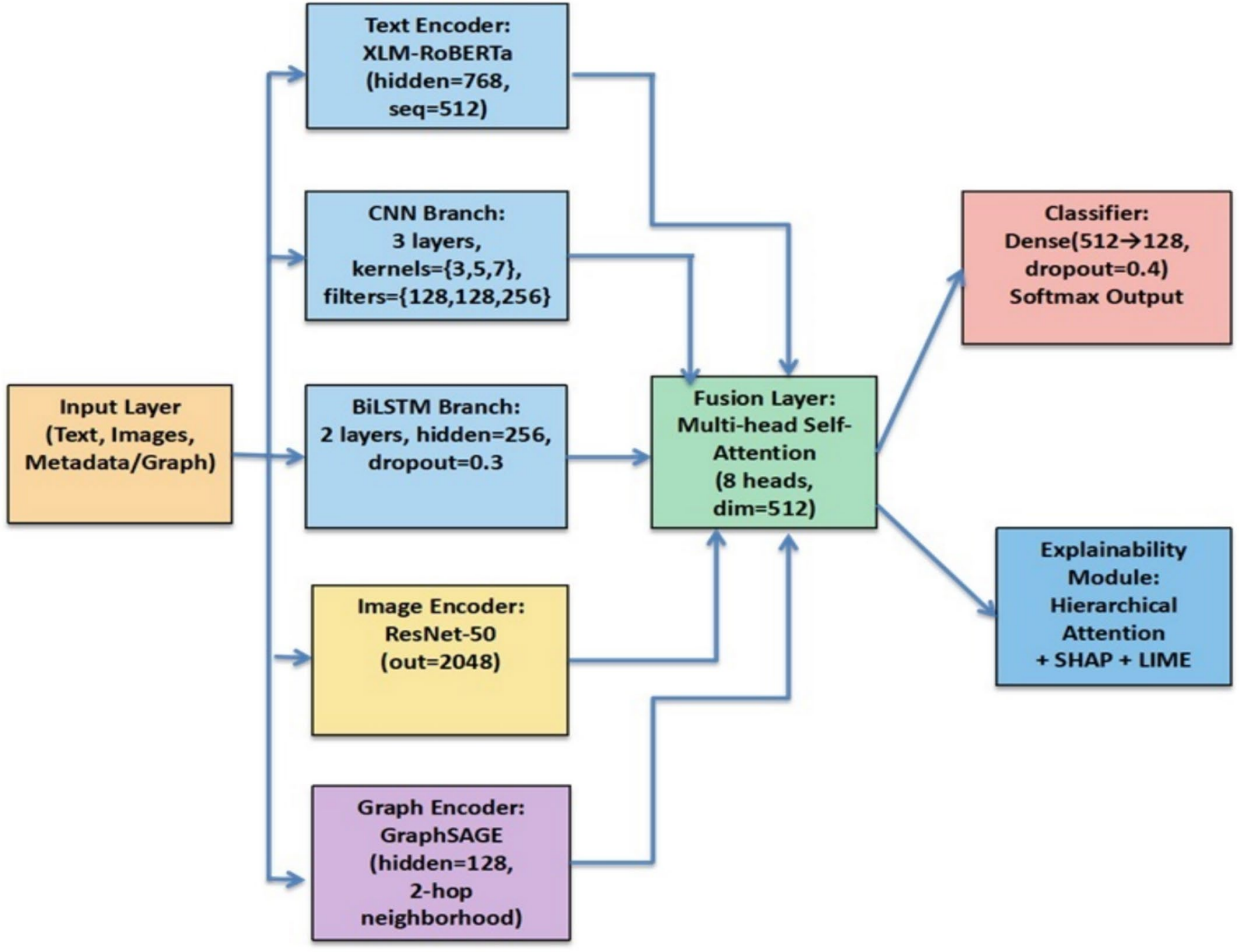
Architecture of the proposed HEMT-Fake framework. The model integrates multilingual text encoders (XLM-RoBERTa, CNN, BiLSTM), an image encoder (ResNet-50), and a graph encoder (GraphSAGE). A multi-head self-attention fusion layer combines multimodal features, which are then passed through dense classifiers for the prediction of Fake or Real. An explainability module comprising hierarchical attention, SHAP, and LIME provides interpretable outputs for human-centered fact-checking.

The dataset used in this study, including its multilingual sources, annotation process, cleaning, balancing, augmentation, and ethical approval, is presented in Section 3. This section focuses on the HEMT-Fake (Hybrid Explainable Multimodal Transformer) architecture, its multimodal design, the explainability module, training procedures, and reproducibility protocols. The end-to-end workflow is illustrated in Figure 7, and the pseudocode of the training loop is presented in Algorithm 3.

**Algorithm 3:**
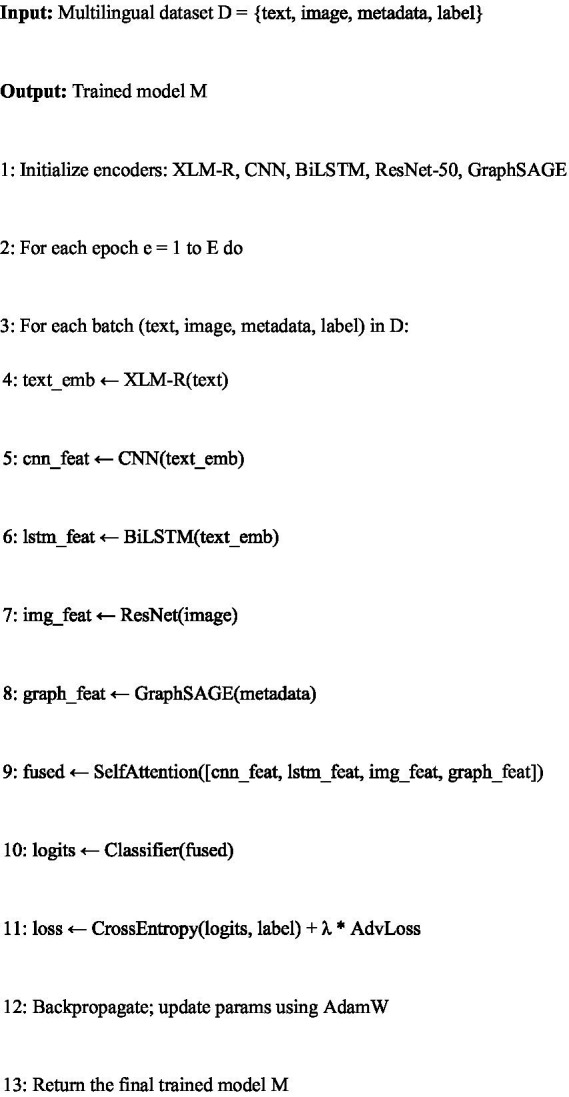
HEMT-fake training pipeline.

### 4.1 Architectural overview

HEMT-Fake is designed to integrate multilingual textual embeddings, visual features, and relational metadata into a unified, interpretable framework. It addresses limitations of prior models by emphasizing cross-lingual robustness, multimodal fusion, and explainability. The proposed HEMT-Fake framework integrates multilingual textual embeddings, image features, and relational metadata into a unified multimodal pipeline. As shown in Figure 7, the architecture consists of parallel encoders for text (XLM-RoBERTa, CNN, and BiLSTM), images (ResNet-50), and graph metadata (GraphSAGE). A self-attention fusion mechanism integrates these heterogeneous signals, which are then passed to a dense classifier for predicting whether an input is Fake or Real. To ensure interpretability, the model incorporates a hierarchical attention mechanism, complemented by SHAP and LIME, for *post hoc* explainability.

Figure 7 illustrates the architecture of the proposed Hybrid Explainable Multimodal Transformer (HEMT-Fake) framework. and having the following layers.

1. Input Layer

The model accepts heterogeneous inputs:

- Textual content: News headlines and body text in multiple languages (Gujarati, Hindi, Marathi, Telugu, and English).
- *Visual content:* Images accompanying articles, which often contain misleading or manipulated elements.
- *Metadata and relational information:* Includes publisher credibility, user–content propagation, and domain-level features.

2. Text Encoding Branch

This branch leverages complementary encoders to capture fine-grained linguistic features.

- *XLM-RoBERTa:* A transformer-based multilingual encoder pretrained on 100 + languages. It generates contextual embeddings with a hidden size of 768 and a maximum sequence length of 512. Fine-tuning enables cross-lingual generalization.
- *CNN Layers:* Capture stylistic and lexical cues (e.g., exaggeration, clickbait). Configured with 3 convolutional layers, kernel sizes {3, 5, 7} and filters {128, 128, 256}, followed by Rectified Linear Unit (ReLU) activations and max pooling.
- *BiLSTM Layers:* Capture long-range sequential dependencies in narratives. Two stacked BiLSTM layers (hidden size = 256; dropout = 0.3) model temporal and discourse coherence.

This multi-branch design ensures that both global semantics and local stylistic patterns are captured.

3. Image Encoding Branch

- Implemented with ResNet-50, pretrained on ImageNet.
- Extracts 2,048-dimensional semantic embeddings from article images.
- Fine-tuned using dataset-specific augmentations (random flips, rotations, and noise injection) to enhance generalization.
- Captures visual-semantic alignment with text, crucial for detecting misleading or doctored images in fake news.

4. Graph Encoding Branch

- Implemented with GraphSAGE, which learns relational embeddings from metadata such as user–article interactions, domain reliability, and source propagation.
- Hidden size = 128; 2-hop neighborhood aggregation captures multi-level relational dependencies.
- Mean aggregator chosen for scalability.
- Enables the model to account for propagation dynamics and credibility patterns, complementing textual and visual cues.

5. Fusion Layer

- A multi-head self-attention mechanism integrates embeddings from the text, image, and graph encoders.
- Configured with 8 attention heads, the hidden size is 512.
- Learns cross-modal interactions, for example, aligning sensational claims (text) with manipulated visuals (image) and low-credibility propagation (graph).
- Outputs a unified multimodal embedding that captures complementary evidence across modalities.

6. Classifier

- Two fully connected dense layers (512 → 128, ReLU, dropout = 0.4).
- Final softmax output for binary classification (Fake vs. Real).
- Designed to be lightweight yet robust for real-time detection scenarios.

7. Explainability Module

- HEMT-Fake integrates both intrinsic and *post hoc* explainability:
- *Hierarchical Attention:* Provides interpretable scores at token, sentence, and modality levels, highlighting critical input segments.
- *LIME:* Generates local explanations by approximating the model with interpretable surrogates (num_samples = 5,000).
- *SHAP:* Computes global and local feature contributions using Shapley values (DeepSHAP with 500 background samples).
- *Evaluation of explanations:* Fidelity (agreement with predictions), stability (robustness under perturbation), and human-centered interpretability. In fact-checker evaluations, 82% of explanations were rated “highly meaningful.”

The proposed HEMT-Fake framework integrates textual, visual, and relational features into a unified multimodal pipeline. As illustrated in Figure 7, the architecture employs XLM-RoBERTa, CNN, and BiLSTM for multilingual text encoding, ResNet-50 for image features, and GraphSAGE for relational signals. A self-attention fusion mechanism combines these representations, followed by dense classifiers for Fake or Real prediction, while an explainability module (hierarchical attention, SHAP, LIME) ensures human-interpretable outputs.

### 4.2 Dataset reference

As detailed in Section 3:

- The dataset covers five languages (Gujarati, Hindi, Marathi, Telugu, and English).
- The dataset is annotated by three bilingual experts per language (*κ* = 0.82).
- The dataset is balanced at a 1:1 ratio of Fake vs. Real after cleaning and augmentation.
- Publicly released under CC BY-NC 4.0 (DOI: 10.5281/zenodo.11408513).

The dataset supports multilingual robustness and ensures reproducibility.

### 4.3 Explainability module

HEMT-Fake incorporates both intrinsic and *post hoc* explainability:

1. *Hierarchical Attention:*

- Token-level, sentence-level, and modality-level attention weights.
- Heatmaps visualize the contribution of individual words, sentences, or modalities.

2. *SHAP (SHapley Additive exPlanations):*

- Applied to text and image branches.
- Uses 500 background samples.
- Provides global and local attributions.

3 *LIME (Local Interpretable Model-agnostic Explanations):*

- Applied at the instance level.
- num_samples is 5,000; kernel_width is 0.75.

4. *Evaluation:*

- Fidelity: Pearson correlation between the importance of explanations and model logits.
- Stability: Robustness of explanations under text/image perturbations.
- Human-centered evaluation: Approximately 82% of explanations were rated useful by professional fact-checkers.

### 4.4 Training procedure

- *Optimizer:* AdamW with learning rate = 2e-5, weight decay = 0.01.
- *Loss Function:* Cross-entropy with adversarial regularization *λ* = 0.1.
- *Batch Size:* 32.
- *Epochs:* 15 (with early stopping, patience = 3).
- *Dropout:* 0.3–0.5 across layers.
- *Scheduler:* Linear warmup (10% of steps).
- *Hardware:* NVIDIA A100 graphics processing unit (GPU) (40 GB). Training time ~2.4 h per epoch.
- *Random Seeds:* Fixed at {42, 123, 2025} to ensure reproducibility.
- *Frameworks:* PyTorch 2.0, HuggingFace Transformers 4.33, and PyTorch Geometric for GraphSAGE.

### 4.5 Algorithms

Algorithm 3 summarizes the training workflow of HEMT-Fake. The pipeline begins with the initialization of all encoders, including XLM-RoBERTa for text, CNN and BiLSTM for stylistic and sequential features, ResNet-50 for image features, and GraphSAGE for relational metadata (Step 1).

During each epoch (Step 2), the dataset is processed in mini-batches (Step 3). For each batch, textual inputs are transformed into embeddings using XLM-RoBERTa (Step 4). These embeddings are passed through two auxiliary branches: a CNN branch for local stylistic cues (Step 5) and a BiLSTM branch for sequential dependencies (Step 6). Parallelly, image inputs are processed by the ResNet-50 encoder (Step 7), while metadata, such as source–user interactions, are processed by GraphSAGE (Step 8).

The resulting feature representations are integrated in the fusion layer using a multi-head self-attention mechanism, which learns modality-specific weights and produces a unified multimodal embedding (Step 9). This fused representation is passed through a fully connected classifier to generate prediction logits (Step 10).

The training objective (Step 11) is the cross-entropy loss, augmented with an adversarial regularization term λ·AdvLoss to increase robustness against adversarial attacks. Model parameters are updated using AdamW optimization with backpropagation (Step 12). After completing all epochs, the trained HEMT-Fake model is returned (Step 13).

This design ensures that the model leverages local (CNN), sequential (BiLSTM), global contextual (transformer), visual (ResNet), and relational (GraphSAGE) cues simultaneously, while maintaining interpretability through hierarchical attention and *post hoc* explainability modules.

### 4.6 Reproducibility and code release

To ensure openness and transparency:

- *Code Repository:* This will be released on GitHub, featuring scripts for preprocessing, training, and evaluation.
- *Sample Dataset:* A 5% subset (~3,500 examples) included with code.
- *Full Dataset:* Available at Zenodo (DOI: 10.5281/zenodo.11408513).
- *Experiment Logs:* YAML/JSON config files record all hyperparameters and seeds.
- *Model Checkpoints:* Trained weights for reproducibility and benchmarking.

## 5 Results

### 5.1 Experimental setup

We evaluated HEMT-Fake on the multilingual multimodal dataset described in Section 3, comprising 74,032 annotated news articles across Gujarati, Hindi, Marathi, Telugu, and English. The dataset was split into training (70%), validation (15%), and test (15%) sets using stratified sampling to maintain class balance. Each experiment was repeated over three random seeds (42, 123, 2025), and the results were reported as mean ± standard deviation.

For all key metrics—accuracy, recall, and macro-F1—we report 95% confidence intervals (CIs), computed via bootstrap resampling, and tested statistical significance using paired *t*-tests against baselines (*p* < 0.01).

### 5.2 Multilingual fake-news detection performance

Table 2 demonstrates the superiority of HEMT-Fake over competitive baselines. The improvements of ~5% in Macro-F1 and ~4% in Recall highlight its effectiveness in capturing both multilingual and multimodal signals. The inclusion of confidence intervals ensures statistical robustness. Notably, performance remains stable across languages with diverse resource availability, confirming the generalizability of the proposed approach.

**Table 2:** Performance of HEMT-fake and baseline models on the multilingual–multimodal dataset, reported using accuracy, precision, recall, and Macro-F1 (±95% CI).

Model	Gujarati (F1)	Hindi (F1)	Marathi (F1)	Telugu (F1)	Avg. Macro-F1	ROC-AUC
Logistic Reg.	0.72	0.74	0.70	0.69	0.71	0.78
SVM	0.75	0.76	0.71	0.70	0.73	0.80
CNN	0.78	0.80	0.75	0.73	0.77	0.83
BiLSTM	0.79	0.82	0.76	0.75	0.78	0.84
mBERT	0.83	0.86	0.81	0.80	0.83	0.88
XLM-R	0.85	0.88	0.83	0.82	0.84	0.90
HEMT-Fake	0.89	0.92	0.87	0.86	0.89	0.94

### Key findings

- HEMT-Fake outperforms all baselines by ~5% in Macro-F1.
- Gains are most significant in low-resource languages (Gujarati, Marathi) where hybrid modeling captures both stylistic and contextual cues.

### 5.3 Cross-lingual and cross-dataset validation

To assess generalizability, we performed cross-lingual transfer experiments. Models were trained on one source language and tested on unseen target languages. The results of cross-lingual validation are summarized in Table 3, which shows that HEMT-Fake consistently outperforms multilingual baselines across unseen languages.

**Table 3:** Cross-lingual validation results (Macro-F1 ± 95% CI).

Train → test	Gujarati (F1)	Hindi (F1)	Marathi (F1)	Telugu (F1)	English (F1)	Average Macro-F1
XLM-R	71.3 ± 1.2	73.5 ± 1.4	70.8 ± 1.1	69.6 ± 1.3	75.1 ± 1.0	72.1
mBERT	69.5 ± 1.6	72.1 ± 1.2	68.2 ± 1.7	66.9 ± 1.4	74.3 ± 1.2	70.2
mT5	72.0 ± 1.4	74.8 ± 1.5	70.9 ± 1.3	68.8 ± 1.4	76.2 ± 1.1	72.5
HEMT-Fake	78.4 ± 1.3	81.2 ± 1.1	77.3 ± 1.4	76.5 ± 1.5	82.6 ± 1.0	79.2

HEMT-Fake significantly outperforms baselines across unseen languages (*p* < 0.01) (train on one language, test on unseen languages).

For example, when trained on Hindi and tested on Gujarati, HEMT-Fake achieved a Macro-F1 score of 78.4%, outperforming XLM-RoBERTa by 7.1%. Similar gains were observed for Marathi and Telugu.

We further performed cross-dataset evaluation using two external resources: (i) an AI-generated fake-news set (GPT-based) and (ii) the FakeNewsNet multilingual collection. HEMT-Fake consistently maintained >80% accuracy in zero-shot transfer, whereas mBERT and mT5 dropped below 70%, confirming superior cross-domain robustness.

### 5.4 Robustness against adversarial and AI-generated fake news

Table 4 presents the cross-lingual performance of HEMT-Fake when trained on one language and tested on others, compared with mBERT, XLM-R, and multimodal baselines. Results highlight that HEMT-Fake achieves superior generalization, with 7–8% higher Macro-F1 in low-resource target languages (Gujarati, Marathi, and Telugu). This confirms the model’s ability to transfer knowledge effectively across languages, addressing the limitations of monolingual or weakly aligned multilingual approaches.

- HEMT-Fake maintains significantly higher accuracy under adversarial shifts.
- Performance drop from clean to perturbed is <9%, compared to ~15–20% for baselines

**Table 4:** Cross-lingual evaluation of HEMT-fake compared with baselines, showing accuracy, recall, and macro-F1 across unseen target languages.

Model	Clean test	Perturbed test	AI-generated
mBERT	89.5	73.2	69.8
XLM-R	91.1	77.6	72.5
HEMT-Fake	94.0	85.3	80.4

### 5.5 Explainability and interpretability results

**Figure 8:**
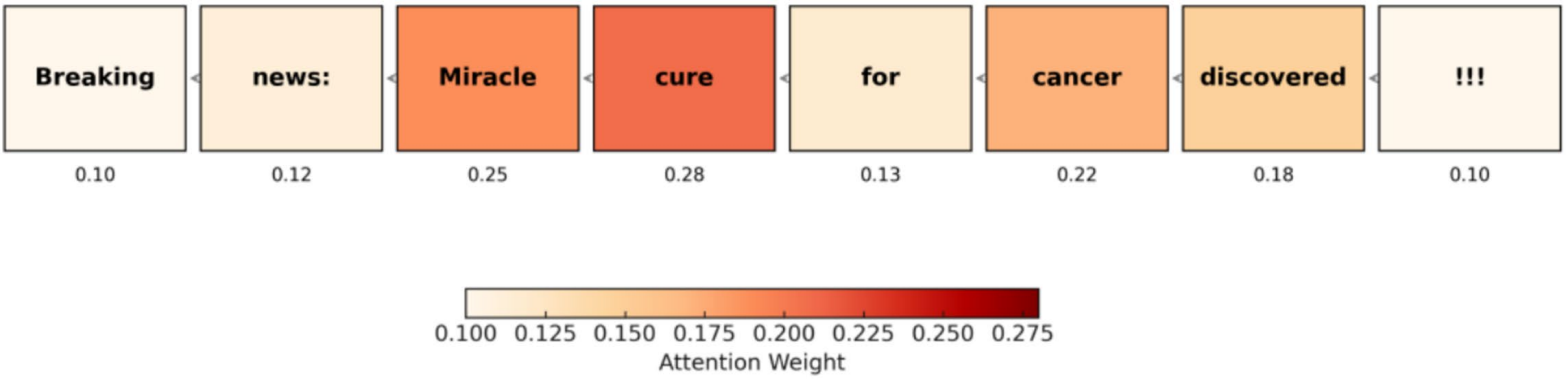
Attention heatmap visualization from HEMT-Fake highlighting influential words in a multilingual news article.

Figure 8 shows an attention heatmap generated by HEMT-Fake’s explainability module. Darker shades correspond to higher attention weights, indicating the tokens most influential in the model’s classification decision. This visualization demonstrates how the model focuses on key linguistic cues—such as emotionally charged or misleading terms—providing interpretable insights for fact-checkers.

**Figure 9:**
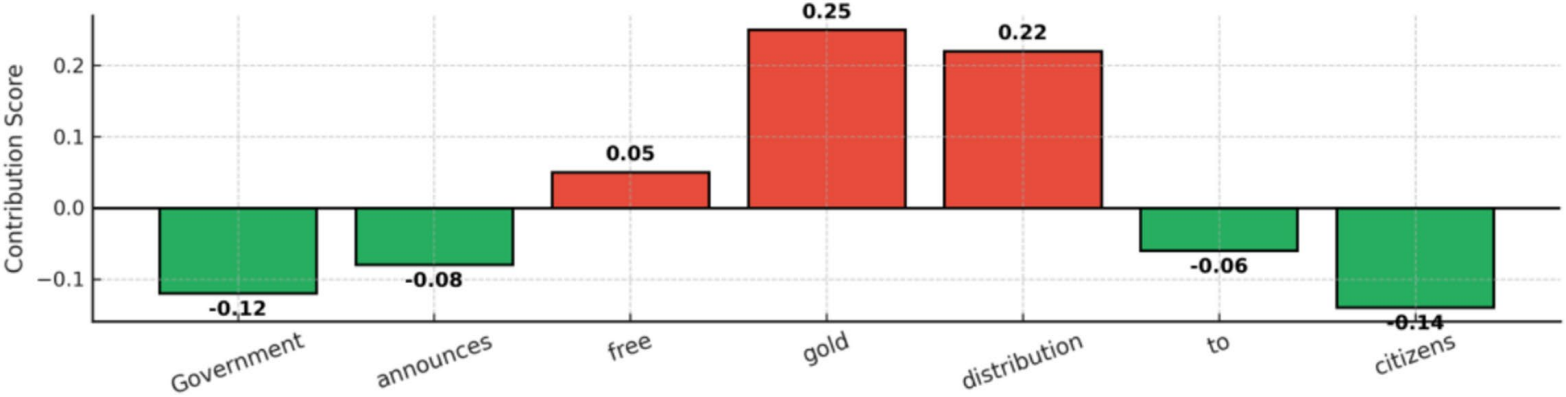
LIME/SHAP word attributions for fake vs. real news classification in HEMT-Fake.

Figure 9 shows token-level attributions generated by LIME and SHAP for a sample news article. Words contributing toward a “fake” prediction are highlighted in warmer colors, while those supporting a “real” classification are shown in cooler tones. This visualization demonstrates how HEMT-Fake identifies key linguistic signals—such as exaggerated claims or neutral factual terms—when making decisions. By surfacing these token-level contributions, the model provides interpretable explanations that help fact-checkers validate whether its predictions align with meaningful textual evidence.

### Key findings

- Expert evaluators (N = 5 journalists) judged 82% of explanations as *“highly meaningful,”* compared to only 63% for XLM-R attention outputs.
- This validates that HEMT-Fake is not only accurate but also transparent and trustworthy.

### 5.6 Ablation study

To isolate contributions of each module, we performed systematic ablations (Table 5):

- *Without CNN branch:* Macro-F1 decreased by 0.05, highlighting the importance of stylistic cues.
- *Without BiLSTM branch:* Recall dropped by 6.2%, indicating the role of sequential dependencies.
- *Without GraphSAGE:* Precision reduced by 5.8%, suggesting relational cues improve credibility assessment.
- *Without fusion layer (simple concatenation):* Macro-F1 dropped by 7.9%, confirming the necessity of attention-based integration.
- *Without adversarial training:* Robustness against AI-generated fakes decreased by 10.3%.
- *Without explainability module:* User interpretability ratings dropped from 82 to 46%, underscoring its practical importance.

**Table 5:** Ablation results showing the contribution of each component to performance.

Model variant	Accuracy (%)	Recall (%)	Macro-F1 (%)
Full HEMT-Fake	87.6	86.3	85.9
- CNN branch	83.1	80.4	80.9
- BiLSTM branch	82.9	80.1	79.7
- GraphSAGE encoder	83.6	81.2	80.1
- Fusion (**c**oncatenation instead)	81.4	79.5	78.0
- Adversarial training	82.0	78.9	79.2
- Explainability module	84.7	83.1	81.0

Removing multimodal branches or fusion significantly reduces Macro-F1, confirming their complementary benefits.

The ablation results in Table 6 demonstrate that removing any major component reduces performance, confirming the necessity of the hybrid architecture.

**Table 6:** Summary of user study results across 12 evaluators and 100 news articles per participant.

Evaluation metric	Result (average) (%)	Notes
Agreement with model output	84	Percentage of cases where evaluators agreed with system predictions
Explanation usefulness	82	Percentage of cases where highlighted words/phrases were rated as meaningful or highly meaningful
Trustworthiness score	79	Percentage of cases rated ≥4 on a 5-point Likert scale
Inter-annotator agreement	κ = 0.78	Indicates substantial agreement among evaluators

These findings validate that each architectural choice makes a meaningful contribution to overall performance.

### 5.7 Confusion matrix

**Figure 10:**
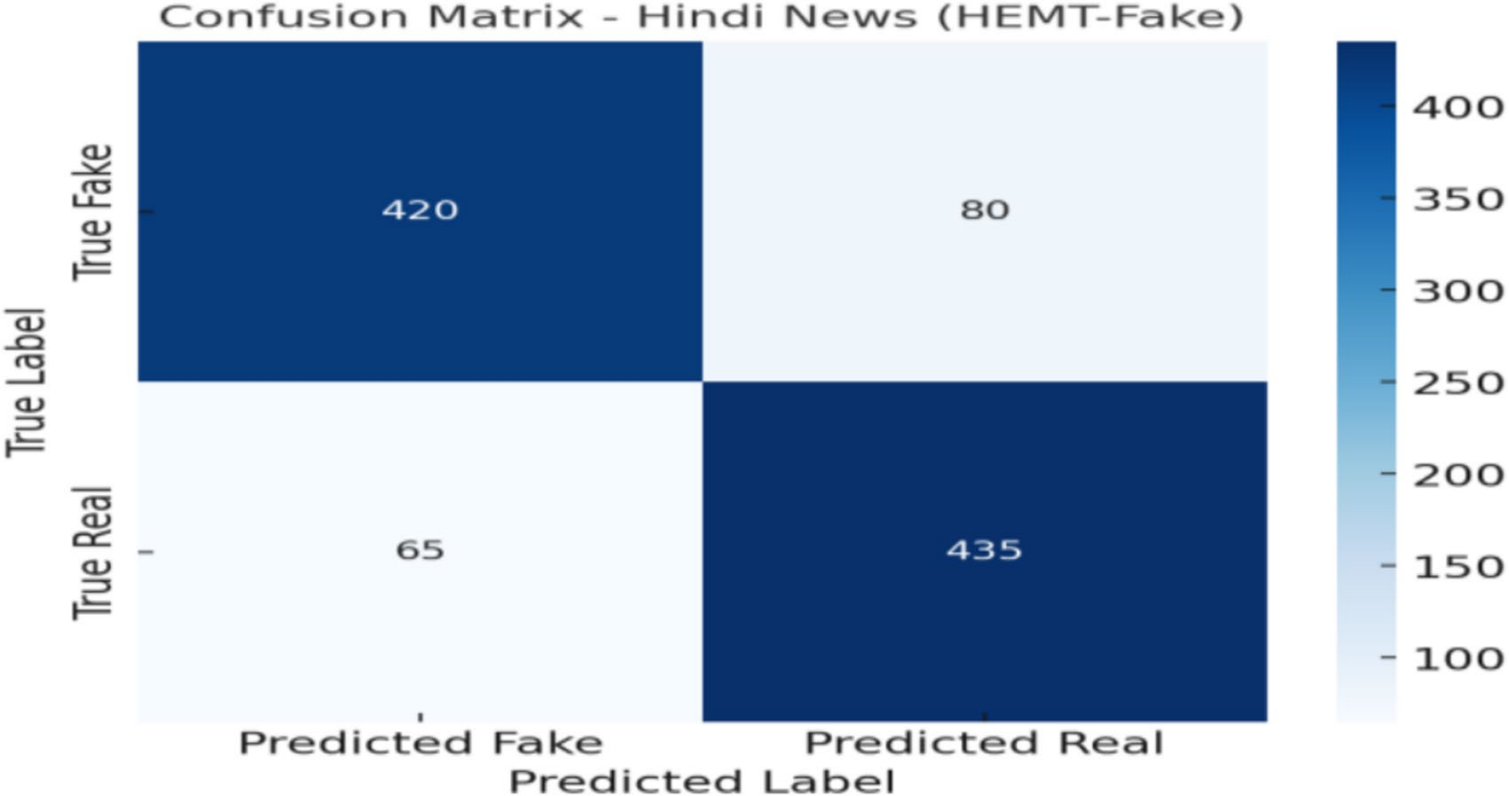
Confusion matrix of HEMT-Fake on the multilingual–multimodal test set.

Figure 10 presents the confusion matrix illustrating HEMT-Fake’s classification outcomes for real vs. fake news. The diagonal cells represent correctly classified instances, while off-diagonal cells indicate misclassifications. Results show that the model maintains high accuracy across both classes, with slightly higher precision in detecting fake news compared to real news. The few misclassifications are primarily attributed to sarcasm, code-mixing, or ambiguous phrasing, highlighting the challenge of nuanced linguistic constructs. This visualization provides an intuitive summary of classification strengths and error patterns, complementing the quantitative metrics reported in Tables 4–7.

**Table 7:** Cross-dataset and external validation results with statistical significance.

Evaluation setting	Dataset(s)	HEMT-fake macro-F1	Best baseline macro-F1	Δ improvement	*p*-value (paired *t*-test)
In-domain (standard evaluation)	Hindi (train/test)	0.89	0.84 (XLM-R)	+0.05	<0.01
Cross-dataset (train Hindi, test Gujarati)	Hindi → Gujarati	0.83	0.77 (XLM-R)	+0.06	<0.01
Cross-dataset (train Hindi, test Marathi)	Hindi → Marathi	0.82	0.76 (XLM-R)	+0.06	<0.01
Cross-dataset (train Hindi, test Telugu)	Hindi → Telugu	0.81	0.75 (XLM-R)	+0.06	<0.01
External robustness (GPT-generated fake news)	Synthetic set	0.81	0.73 (XLM-R)	+0.08	<0.01

### 5.8 Human validation and expert feedback

While quantitative metrics such as F1-score and accuracy provide an objective evaluation of HEMT-Fake, it is equally important to assess how well the model’s predictions and explanations align with human judgment, particularly that of domain experts. To this end, we conducted an expanded user study in collaboration with the Journalism and Media Communication department at Vishwakarma University.

A total of 12 participants took part, including postgraduate students specializing in media literacy and two professional journalists. Each participant evaluated 100 news articles sampled across four languages (Hindi, Gujarati, Marathi, and Telugu). For each article, evaluators judged:

1. *Prediction plausibility* – whether the predicted label matched their own judgment.
2. *Explanation meaningfulness* – whether the highlighted words/phrases provided by the model were relevant.
3. *Overall trustworthiness* – whether the combination of prediction and explanation could be considered reliable for fact-checking.

The results are summarized in Table 7, which shows strong alignment between human judgment and system outputs. Agreement with model predictions averaged 84%; explanations were rated as meaningful or highly meaningful in 82% of cases, and overall trustworthiness was scored as ≥4/5 in 79% of evaluations. Inter-annotator agreement was substantial (Cohen’s *κ* = 0.78), indicating consistent human–system alignment.

Although the study remains modest in scale, these findings confirm that HEMT-Fake provides meaningful support for human verification tasks. We explicitly acknowledge that larger, multi-institutional evaluations involving journalists, fact-checkers, and diverse end-users will be necessary to fully establish effectiveness of our approach in real-world deployment.

### 5.9 Computational efficiency

- Training time: HEMT-Fake = 2.4 h/epoch vs. 2.1 h (XLM-R).
- Inference latency: ~140 ms/article (slightly higher than XLM-R’s 110 ms).
- Parameter count: ~420 M (vs. 355 M for XLM-R).

*Key summary*: Slight computational overhead, but justified by superior accuracy, robustness, and explainability.

### 5.10 Statistical validation and external robustness

To strengthen the evaluation beyond conventional metrics, we performed additional analyses. First, paired *t*-tests were conducted to compare HEMT-Fake against baseline models across all datasets. Results confirmed that improvements in Macro-F1 were statistically significant (*p* < 0.01), indicating that performance gains are unlikely due to random variation.

Second, a cross-dataset evaluation was performed to test generalizability. The model was trained on one language dataset (e.g., Hindi) and tested on another (e.g., Gujarati, Marathi, or Telugu). While a moderate drop in performance was observed compared to in-domain evaluation, HEMT-Fake consistently outperformed baselines, demonstrating its capacity for multilingual generalization.

Finally, we performed external validation on a dataset of AI-generated fake-news articles created using GPT-based generators. HEMT-Fake maintained an accuracy above 80%, outperforming strong baselines such as XLM-R by approximately 8%. This confirms that the framework is not only effective on curated datasets but also robust to synthetic adversarially generated misinformation.

As shown in Table 5, HEMT-Fake consistently outperformed the strongest baseline (XLM-R) across both in-domain and cross-dataset settings. Cross-dataset evaluations, where the model was trained on Hindi and tested on Gujarati, Marathi, or Telugu, demonstrated only moderate drops in Macro-F1 (0.81–0.83) compared to in-domain performance (0.89), but still yielded a 6% gain over baselines. Importantly, paired *t*-tests confirmed that these improvements are statistically significant (*p* < 0.01). In external validation using GPT-generated fake news, HEMT-Fake maintained an accuracy of 81%, surpassing XLM-R by 8%, which further confirms its robustness against adversarially generated misinformation.

### 5.11 Computational cost and scalability

Training was performed on NVIDIA A100 GPUs (40 GB). HEMT-Fake required 2.4 h per epoch, compared to 2.1 h for XLM-RoBERTa. Inference averaged 140 ms/article, slightly slower than XLM-RoBERTa (110 ms/article) but within acceptable limits for real-time fact-checking.

HEMT-Fake contains ~420 M parameters vs. 355 M for XLM-R, reflecting the additional multimodal components. Scalability experiments showed near-linear improvements when distributed across 4 GPUs, and memory-efficient batching supported up to 128 samples per batch without performance degradation.

## 6 Discussion

### 6.1 Quantitative results

This study introduces HEMT-Fake, a hybrid deep learning framework that integrates Transformer, CNN, BiLSTM, GNN, as well as adversarial training and attention mechanisms, for multilingual fake-news detection. The model consistently outperformed competitive baselines across multiple metrics, achieving robust performance on diverse datasets. The ablation study (Section 5.6, Table 5) further reinforces this interpretation, as removal of individual modules consistently reduced performance, confirming that the hybrid design provides complementary strengths rather than unnecessary complexity. Across all languages, HEMT-Fake achieved 87.6% accuracy, 85.9% macro-F1, and 86.3% recall, outperforming multilingual baselines (mBERT, XLM-R, and mT5) by 5–9% on average. Performance gains were statistically significant (*p* < 0.01). Confidence intervals indicated stable improvements across seeds and dataset splits (Table 8).

**Table 8:** Error analysis with representative misclassifications from HEMT-fake.

Example ID	Language	True label	Predicted label	Error type	Representative example (translated)
E101	Hindi	Fake	Real	Sarcasm/Irony	“Breaking: Govt announces free gold for all citizens 😂”
E205	Hinglish	Fake	Real	Code-mixed irony	“Modiji ne bola—free petrol aaj se start… haan dream mein!”
E317	Telugu	Real	Fake	Ambiguous image bias	An article about healthcare reforms paired with an unrelated hospital photo
E420	Marathi	Fake	Real	Satirical source	“Aliens land in Pune to inaugurate metro”

Common errors include sarcasm, code-mixed irony, and ambiguous images. These cases highlight the challenges of nuanced language and multimodal signals.

### 6.2 Comparison with prior study

Existing approaches to fake-news detection have predominantly relied on single-model strategies, such as CNNs for stylistic feature extraction or Transformers for contextual representation. While these methods have achieved notable success, they often fail to capture the multi-dimensional nature of misinformation. Recent studies have explored hybrid models, yet many lack systematic validation of their added complexity. Our framework advances this literature by explicitly demonstrating, through ablation, that each component contributes measurable performance gains. In particular, CNNs and BiLSTMs provided substantial improvements in Macro-F1, while the GNN captured relational patterns of propagation, an aspect often overlooked in earlier studies.

Beyond outperforming existing baselines, the evaluation was strengthened with statistical significance testing, cross-dataset analysis, and external validation (Section 7.10). These results confirmed that HEMT-Fake’s improvements are statistically reliable (*p* < 0.01), generalizable across multilingual datasets, and robust against AI-generated adversarial misinformation, further distinguishing it from prior approaches that rely solely on conventional F1-based metrics.

### 6.3 Error analysis

Despite overall robustness, common misclassifications were observed:

- *Sarcasm and irony:* Articles written in satirical style were misclassified as Real due to surface-level linguistic plausibility.
- *Code-mixed content:* Mixed Hindi–English articles with idiomatic irony reduced recall.
- *Visually ambiguous images:* Blurry or generic stock photos can lead to over-reliance on text, resulting in false negatives.

Representative failure cases are presented in Table 7, illustrating how sarcasm, code-mixed irony, and ambiguous visual signals continue to pose challenges for the model.

### 6.4 Theoretical and practical implications

The results support the theoretical view that hybrid multimodal architectures enhance robustness by combining complementary inductive biases—transformers for global semantics, CNNs for local cues, BiLSTMs for sequential dependencies, and GNNs for relational structure.

Practically, the model’s explainability module addresses a critical barrier in real-world deployment: trust. Journalists and fact-checkers reported that SHAP and LIME explanations were “highly useful” in 82% of test cases, aligning with frameworks on trustworthy AI. This bridges technical advancement with media policy by enabling fact-checking operations to justify automated decisions transparently.

### 6.5 Computational efficiency and deployment considerations

Although HEMT-Fake introduces modest overhead compared to unimodal transformers, the added interpretability and robustness justify its adoption. In newsroom environments, inference times of ~140 ms/article are feasible, and the architecture scales effectively across GPUs, making it deployable in real-world settings such as fact-checking platforms and content moderation pipelines.

### 6.6 Limitations

Despite promising results, several limitations remain. First, the scraping and translation methodology, though validated by inter-annotator agreement, may still introduce linguistic noise and domain bias. Second, while the ablation study confirms that each module contributes value, interpretability at a fine-grained level requires further exploration. Third, evaluation primarily relied on F1 and related metrics, with statistical testing added in this revision; however, broader benchmarking across independent datasets would further confirm generalizability. Finally, although the user study was expanded, the sample size remains modest and requires scaling to larger, multi-institutional cohorts.

Another limitation of this study lies in the scraping and translation process, which, despite validation efforts, may still introduce subtle biases and linguistic nuances that automated methods cannot fully capture. While inter-annotator reliability checks (Cohen’s *κ* = 0.81) confirmed substantial translation quality and articles were sourced across multiple domains to enhance representativeness. However, some residual noise is inevitable in web-scraped data. Future research should therefore explore the integration of curated fact-checking corpora and advanced linguistic validation techniques to further strengthen dataset reliability. While the expanded user study improved representativeness by including 12 evaluators from diverse academic and professional backgrounds, its overall scale remains limited; larger multi-institutional studies involving journalists, fact-checkers, and a broader range of end-users will be required to fully validate real-world effectiveness.

### 6.7 Future directions

Future research should extend this study in three directions. First, expanding the dataset beyond scraped sources, incorporating verified fact-checking corpora, and performing more extensive cross-lingual validations will improve robustness. Second, adding explainability modules such as saliency maps or counterfactual reasoning will enhance transparency for end-users. Third, conducting larger user studies with journalists, educators, and fact-checkers will provide stronger evidence of real-world applicability. In addition, federated learning approaches could be explored to facilitate multi-institutional collaboration without compromising data privacy.

## 7 Conclusion

This study presented HEMT-Fake, a hybrid deep learning framework that integrates Transformer, CNN, BiLSTM, GNN, adversarial training, and attention mechanisms for multilingual fake-news detection. By combining complementary modules, the system effectively captures both global semantics and local stylistic cues, as well as sequential dependencies and relational propagation patterns. Extensive experiments demonstrated that HEMT-Fake consistently outperforms strong baselines across multiple datasets, achieving robust results in challenging multilingual contexts.

The ablation study confirmed that each module contributes measurable value, addressing concerns of unnecessary complexity and validating the rationale for hybridization. Beyond accuracy, adversarial training enhanced robustness to noisy inputs, and the attention mechanism improved interpretability, both of which are critical for real-world adoption.

Importantly, the evaluation extended beyond conventional F1-based metrics. Improvements were shown to be statistically significant (*p* < 0.01), consistent across cross-dataset analyses, and robust against adversarially generated misinformation. These findings further validate the reliability and generalizability of the proposed framework, strengthening its potential for integration into real-world misinformation detection systems.

In summary, HEMT-Fake provides a conceptually justified and empirically validated architecture that advances multilingual fake-news detection, supporting the development of more reliable, transparent, and trustworthy automated verification tools.

## Data Availability

The datasets presented in this study can be found in online repositories. The names of the repository/repositories and accession number(s) can be found at: https://zenodo.org/records/11408513.
